# Guidelines for gene and genome assembly nomenclature

**DOI:** 10.1093/genetics/iyaf006

**Published:** 2025-01-15

**Authors:** Ethalinda K S Cannon, David C Molik, Adam J Wright, Huiting Zhang, Loren Honaas, Kapeel Chougule, Sarah Dyer

**Affiliations:** USDA Agricultural Research Service—Corn Insects and Crop Genetics Research Unit, Crop Genome Informatics Lab, 819 Wallace Rd, Ames, IA 50012, USA; USDA Agricultural Research Service—Arthropod-borne Animal Diseases Research Unit, Center for Grain and Animal Health Research, 1515 College Avenue, Manhattan, KS 66502, USA; Ontario Institute for Cancer Research, Adaptive Oncology, Ontario Institute for Cancer Research, 661 University Ave Suite 510, Toronto, ON M5G 1M1, Canada; Washington State University—Department of Horticulture; USDA Agricultural Research Service—Physiology and Pathology of Tree Fruits Research Unit, Physiology and Pathology of Tree Fruits Research Unit, 1104 N. Western Ave., Wenatchee, WA 98801, USA; USDA Agricultural Research Service—Physiology and Pathology of Tree Fruits Research Unit, Physiology and Pathology of Tree Fruits Research Unit, 1104 N. Western Ave., Wenatchee, WA 98801, USA; Cold Spring Harbor Laboratory—Ware Laboratory, Cold Spring Harbor Laboratory, 1 Bungtown Rd, Cold Spring Harbor, NY 11724, USA; EMBL-EBI—Non-Vertebrate Genomics Team, European Molecular Biology Laboratory, European Bioinformatics Institute, Wellcome Trust Genome Campus, Cambridge CB10 1SD, UK

**Keywords:** genome, gene model, assembly, nomenclature, database

## Abstract

The rapid increase in the number of reference-quality genome assemblies presents significant new opportunities for genomic research. However, the absence of standardized naming conventions for genome assemblies and annotations across datasets creates substantial challenges. Inconsistent naming hinders the identification of correct assemblies, complicates the integration of bioinformatics pipelines, and makes it difficult to link assemblies across multiple resources. To address this, we developed a specification for standardizing the naming of reference genome assemblies, to improve consistency across datasets and facilitate interoperability. This specification was created with FAIR (Findable, Accessible, Interoperable, and Reusable) practices in mind, ensuring that reference assemblies are easier to locate, access, and reuse across research communities. Additionally, it has been designed to comply with primary genomic data repositories, including members of the International Nucleotide Sequence Database Collaboration consortium, ensuring compatibility with widely used databases. While initially tailored to the agricultural genomics community, the specification is adaptable for use across different taxa. Widespread adoption of this standardized nomenclature would streamline assembly management, better enable cross-species analyses, and improve the reproducibility of research. It would also enhance natural language processing applications that depend on consistent reference assembly names in genomic literature, promoting greater integration and automated analysis of genomic data. This is a good time to consider more consistent genomic data nomenclature as many research communities and data resources are now finding themselves juggling multiple datasets from multiple data providers.

## Introduction

Over the past 2 decades, we have seen a transformation in the technologies used to generate and assemble genomes. High-quality genome assemblies are now possible even for species with large, complex, or under-studied genomes (Giani *et al*. 2020; Chapman *et al*. 2022). As a result, we have moved from 1 or no genome assemblies available to researchers studying a specific species, to an era of pan-genomics, where multiple high-quality assemblies are being generated for a species routinely. Such a data-rich environment comes with exciting problems to be solved (Sherman and Salzberg 2020; Li *et al*. 2022). To take advantage of this new data, the seemingly simple problem of genome and gene model nomenclature must be addressed to identify assemblies, unambiguously link gene model identifiers to their annotation dataset and source assembly, and enable analysis of multiple datasets within and across species. Consistent, unambiguous nomenclature will help to make data FAIR (Findable, Accessible, Interoperable, and Reproducible) (Wilkinson *et al*. 2016). In contrast, classical gene names and symbols are generally better addressed, for example in wheat (Boden *et al*. 2023) and human (Bruford *et al*. 2020).

The AgBioData Research Coordination Network is a consortium of agriculturally relevant databases, focused on solving common agricultural research data problems (Harper *et al*. 2018; https://agbiodata.org). To address the need for consistent and unambiguous genomic data nomenclature, a Working Group under the auspices of AgBioData was formed and tasked with generating best practices for naming genome assemblies and predicted gene models. The diversity of organisms studied by members of the consortium, the community's willingness to adopt changes, and community recognition of the importance and lack of gene model and genome nomenclature recommendations, make the AgBioData community an ideal proving ground for genome nomenclature practices.

There are several primary data repositories, including some that specialize in genomic data for any species, which collect metadata for datasets and provide unique accessions. The most-used primary genomic data repositories are members of the International Nucleotide Sequence Database Collaboration (INSDC; https://www.insdc.org/) consortium, which includes GenBank (https://www.ncbi.nlm.nih.gov/genbank), the European Nucleotide Archive (ENA; https://www.ebi.ac.uk/ena), and the DNA Data Bank of Japan (DDBJ; https://www.ddbj.nig.ac.jp/). Primary data repositories typically do not restrict nomenclature beyond some simple constraints including limits on length, permitted characters, and rules for not including certain terms, like taxonomic names, in either assembly or gene names, which is the case for INSDC repositories. This leaves the establishment of nomenclature rules to the research communities themselves. We have worked with representatives from the INSDC to ensure that the naming scheme described here is in keeping with INSDC policies (personal communications, 9/24).

Some communities we worked with while developing this nomenclature recommendation include The Arabidopsis Information Resource (TAIR; https://www.arabidopsis.org/ Reiser et al., 2024), and the Genome Database for Rosaceae (GDR; https://www.rosaceae.org/ ; Jung et al., 2019). In both cases, the communities had existing nomenclature guidelines, but have found they are no longer sufficient for current data and research needs.

### Why nomenclature rules are important

Names identify data. Biologists often want information encoded in names to enable quick identification of assemblies and gene models, for example, species, cultivar/breed/strain, chromosome, and sequential numbering along chromosomes, but also do not want long identifiers (see AgBioData discussion results in *Materials and methods* and Supplementary File 1) Although numeric identifiers are a great way to uniquely identify data, for example, in computational analyses, they are not as informative as names that contain minimal metadata. This is particularly true when each assembly follows a consistent naming convention.

Adopting a balance between human-readable and machine-readable nomenclature addresses a broad range of research needs. Researchers can quickly identify genome datasets, and analysis pipelines do not need special case handling of multiple naming schemes. Also important is that gene model identifiers contain information linking them to their annotation and source assembly, so gene models reported in papers can be easily linked to their assembly versions. In another example, sequential numbering along chromosomes may immediately alert researchers to potential split gene models, tandem arrays, or other significant positional information that would otherwise take additional steps to resolve numeric identifiers to genome locations. Consistent nomenclature also benefits natural language processing and machine learning analyses of research papers and datasets.

An example of the confusion that can arise when there are no community standards for nomenclature can be seen in the names of Arabidopsis assemblies held by GenBank. Examples include: TAIR10.1, Tanz-1.10024.PacbioHiFiAssembly, ASM2311539v1, 6137, AT5784.Ty-1.PacBio, AtA1-141.20181003, Arabidopsis_thaliana_Ler, Ath.Ler-0.MPIPZ.v1.0, ddAraThal4.1, Ler Assembly, ONTmin_IT4, and others. The Arabidopsis community is now addressing this problem, as described below, in *Materials and methods*.

The proposed nomenclature enables FAIR data as follows (for a full checklist to enable FAIR in naming, and an assessment of how well the later proposed naming scheme enables data to fulfill this checklist, see Supplementary File 2).

- Findability: Make relevant genome assemblies easier to find with unambiguous naming. Ideally, this means that a name contains minimal metadata information which can be used to filter data objects. It follows that names should also be unique.
- Accessibility: To enable data to be more accessible and retrievable, the nomenclature rules should be well documented. Validation tools are likely helpful for assessing names, and provenance data should be attached to the names themselves.
- Interoperability: Names should be both human- and machine-readable, and should minimize the need to wrestle with multiple nomenclatures.
- Reuse: Unambiguous naming supports genome and gene model sharing and reuse.

### Challenges to adopting a nomenclature

For any established research community, adopting and enforcing a nomenclature, especially if replacing an existing nomenclature, can be challenging (Hollmann *et al*. 2020). Likewise, data resources that handle large quantities of genomic data across multiple species and genera may be resistant to change, possibly due to internal pipelines that expect an existing nomenclature, concerns about disrupting user experience, internal policies to not enforce nomenclature rules, or perceived difficulty of adopting and enforcing a nomenclature.

To solve adoption problems, it may be necessary to permit some flexibility in the nomenclature, allowing for naming practices that may be unique and necessary for a specific research community. Existing identifiers could be listed as synonyms via metadata, or if deemed necessary by the community, appended to identifiers that otherwise follow some or all of the recommendations described in *Results and discussion*.

Some factors that can make the adoption and enforcement of a nomenclature more feasible include a new organism research community or a complete overhaul of a representative reference genome and annotation; and the existence of a primary data resource or closely integrated set of data resources that can enforce nomenclature rules. An example is MaizeGDB (Woodhouse et al. 2021), the primary resource for maize genetic and genomic data, which has been largely successful in enforcing its nomenclature rules for genomic data (MaizeGDB 2021).

### Examples of adoption

Adoption of the proposed nomenclature may be partial or incremental due to the need to balance researchers' expectations against the need for change. Researchers find changes in nomenclature to be quite challenging, especially when it happens partway through a specific research project.

One data resource that has adopted the nomenclature rules presented in this paper is the Genome Database for Rosaceae (GDR; https://www.rosaceae.org/nomenclature/genome), which houses genomic resources for multiple economically important species in the rose family. In recent years, there has been a notable increase in the deposition of phased, cultivar-specific genome assemblies. Consequently, the existing gene model naming guidelines, producing identifiers like Fv.01g000010 (species *Fragaria vesca*, chromosome 1, gene model number 000010), initially proposed by the community a decade ago, needs revision. The pome fruit research community, which includes apples and pears within the Rosaceae family, has proposed a new gene naming convention (e.g. Maldo.hc.v1a1.ch10A.g00001.t1; Khan *et al*. 2022). This updated convention incorporates species “Maldo”, cultivar “hc”, chromosome number “chr10”, haplome “A”, and the versions of the assembly and annotation (v1a1). This nomenclature has been followed by 2 other genomes (‘d’Anjou’ pear: Yocca *et al*. 2024; ‘WA 38' apple: Zhang *et al*. 2024) and is being adopted by GDR with modifications—for example, the use of the ToLID “drMalDome” in place of “Maldo”—for the refinement of the guidelines (GDR, c2010-2024). GDR works with data providers to ensure proper nomenclature, in some cases, prepending assembly information to gene models if lacking in the original dataset. GDR feels that the new recommendations are working for their community. Several databases that are closely related to GDR are also using the GDR nomenclature for new genome datasets. These include the Genome Database for Vaccinium (https://www.vaccinium.org), the Citrus Genome Database (https://www.citrusgenomedb.org), the Pulse Crop Database (https://www.pulsedb.org), and CottenGen (https://www.cottongen.org).

The Arabidopsis Information Resource (TAIR) is in the process of revising genomic nomenclature rules, balancing the need to support the historic naming conventions for Arabidopsis genomic data with the need to revise naming conventions to reflect the current data and research landscape. The Arabidopsis research community has been producing genomic data for decades, so introducing a new nomenclature is challenging, and presents limitations on how much names can change. In the upcoming ‘Col-0’ reannotation, the genome assembly name “At3702.Col-0.Col-CC.v2” follows this convention: <taxon><variety><ID><version>, where “taxon” consists of the first letter of the genus and the first letter of the species followed by the NCBI taxon id; “variety” is the short cultivar name; “ID” is a short identifier for the assembly; and “version” is the letter “v” followed by the version number. In this example, ‘At3702' identifies *Arabidopsis thaliana*, where “3702” is its taxonomic accession, ‘Col-0’ is the variety, ‘Col-CC’ is an abbreviation for “Col-0 Community Consensus”, and the version number is 2. The genome annotation name is formally At3702.Col-0.Col-CC.v2.1, or <assembly name>.<annotation version>. For convenience, TAIR will refer to the genome assembly as Col-CC.v2 and to the genome reannotation on that assembly as TAIR12. Individual loci are referred to using the AGI (Arabidopsis Genome Initiative) format (e.g. At1g01010) that was established in 1999 with the annotation version appended for specificity: At1g01010_TAIR12 and gene models using the style At1g01010.1_TAIR12.

## Materials and methods

The AgBioData genomic nomenclature Working Group members manage and conduct research with genomic data from a range of agriculturally important species and have experienced first-hand the challenges of working with nonstandard genomic nomenclature. The Working Group surveyed existing nomenclature rules from other research communities, such as human gene naming (Bruford *et al*. 2020); genes and other structural elements for wheat (Boden *et al*. 2023); and nomenclature used by the Vertebrate Genomes Project (Rhie *et al*. 2021; https://vertebrategenomesproject.org), paying particular attention to the Tree of Life identifiers (ToLID, n.d, https://id.tol.sanger.ac.uk/). We found very few instances of genomic nomenclature specifications, so we also surveyed examples of assembly and gene model identifiers used in various research communities (Tables 1 and 2) and examined the few instances of formal genomic nomenclature guidelines we were able to find.

**Table 1.** Examples of gene model identifiers.

Examples	Species	Assembly version	Accession	Subgenome	Chromosome	Entity	ID #	Annot. version
C01p010030.1_BnaDAR http://brassica.info/tools/data_standards.html	B na (*Brassica napus*)		DAR	C	01	p	010030	.1
HORVU.MOREX.r3.6HG0548430 https://plants.ensembl.org/Hordeum_vulgare/Gene/Summary?g=HORVU.MOREX.r3.6HG0548430;r=6H:21859180-21859461;t=HORVU.MOREX.r3.6HG0548430.1	HOR VU (*Hordeum vulgare*)	r3	MOREX		6H	G	0548430	
TraesCS3D02G273600 https://plants.ensembl.org/Triticum_aestivum/Gene/Summary?gTraesCS3D02G273600;r=3D:379535906-379539827	Tr aes (*Triticum aestivum*)		CS	D	3	G	273600	02
Vitvi18g12230 10.1186/1471-2164-15-1077	Vit vi (*Vitis vinifera*)				18	g	12230	
**Zm00001eb000050** https://documents.maizegdb.org/nomenclature/maize_assembly_nomenclature_2021.pdf	**Z m (*Zea mays*)**	**e**	**00001**				**000050**	**b**
(SORBI_3)/(Sobic.)006G095600 https://www.ncbi.nlm.nih.gov/pmc/articles/PMC1449135/	SORBI_3/Sobic(*Sorghum bicolor*)				006	G	0965600	

Bold font indicates communities with gene model nomenclature rules or recommendations. The non-bolded examples show 1 of multiple nomenclature patterns used within the community.

**Table 2.** Examples of genome assembly identifiers.

Examples	Species	Assembly version	Accession/cultivar	Sequencing group/method	Haplotype
**Maldo.hc.v1.ch10A** https://www.rosaceae.org/nomenclature/genome	***Malus domestica***	**v1**	**hc (Honeycrisp)**		**A**
AST_PRJEB5043_v1 https://plants.ensembl.org/Brassica_napus/Info/Index	*Brassica napus*	v1	PRJEB5043		
Glycine max Wm82.a6.v1	*Glycine max*	a2	Wm82		
MorexV3_pseudomolecules_assembly https://plants.ensembl.org/Hordeum_vulgare/Info/Annotation/	*Hordeum vulgare*	v3	Morex		
C_sonorensis_v2_redundans https://www.ncbi.nlm.nih.gov/datasets/genome/GCA_900258525.3/	*Culicoides sonorensis*	v2		Redundans	
**Zm-B73-REFERENCE-NAM-5.0** https://documents.maizegdb.org/nomenclature/maize_assembly_nomenclature_2021.pdf	***Zea mays***	**5.0**	**B73**	**NAM consortium**	

Bold font indicates communities with assembly nomenclature rules or recommendations. The non-bolded examples show 1 of multiple nomenclature patterns used within the community.

The Working Group also discussed ideas for an ideal genomic data nomenclature with AgBioData consortium members. One outcome of the discussion that was recorded via Google Forms (Supplementary File 1) is that genome assemblies and gene models should be given names that are both human- and machine-readable, and that nomenclature rules should provide unambiguous identification across species and clades (note, however, that given the large variability in the naming and abbreviations of species, clades, strains, cultivar, etc., truly unambiguous identification cannot be guaranteed.) Because different groups may sequence the genomes of different individuals within the same cultivar/strain, indicating which group did the work is also important for disambiguating genome assemblies.

An alternative approach to encoding complete information in the name or expecting to enforce naming conventions on a disparate group of data providers is taken by the Legume Information System Datastore (LIS; https://data.legumeinfo.org ; Berendzen et al., 2021). Datasets are grouped into “collections” similar to the U.S. Library of Congress BagIt File Packaging Format (https://www.ietf.org/rfc/rfc8493.txt). Each collection name is unique within the project datastore and is included in the contained data- and metadata files. Collection names have a human-readable component consisting of a strain or study identifier, an abbreviated data type and version, and a unique identifier or “key”. The unique collection key is included as part of each filename within the collection, associating each file with the metadata for that collection. Within the collection (a directory), structured human- and machine-readable README and MANIFEST files indicate the provenance of each file, the collection contents, and associated metadata such as citations and other identifiers and repositories. Each collection is organized by genus and species and broad data type. For example, the soybean genome assembly for strain (accession) ‘Williams 82’ is contained within a collection named Wm82.gnm6.S97D, within a directory structure of Glycine/max/genomes. Thus, the Legume Information System group applies naming and organizational patterns consistent within the project, while maintaining provenance and a record of other filenames as part of the metadata.

## Results and discussion

Based on an assessment of existing genome and gene model nomenclatures across agricultural species and through discussions with partners from the AgBioData community, we have identified the following key elements for generating standardized assembly and gene model names which are both human- and machine-readable.

### Assembly identifiers

**Fig. 1.**
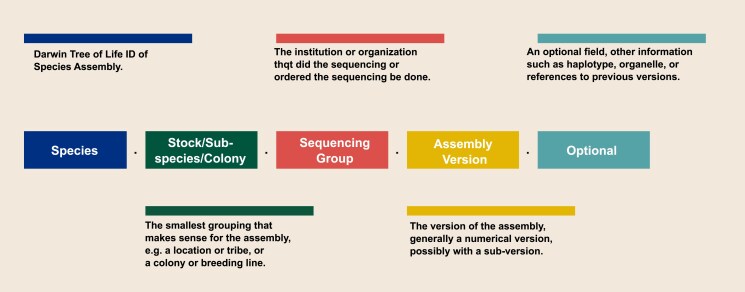
Computationally legible identifiers that are also human-readable. The fields of the names are separated by periods. Each name contains at least 4 fields—species, individual, project group, version—with an optional fifth field to for additional information that may be required for an unambiguous identifier.

The proposed format for assembly identifiers is shown in Fig. 1.

Species: The Tree of Life project provides a self-service system to find or generate unambiguous species identifiers, described in detail at the website, https://id.tol.sanger.ac.uk, as follows:

- A lower-case letter for the high-level taxonomic rank and a lower-case letter for the clade (see clade prefix assignments below). Only 1 letter is used for vertebrates (VGP legacy);
- One upper- and 2 lower-case letters for genus; and
- One upper- and 3 lower-case letters for species (1 upper- and 2 lower-case for vertebrates, VGP legacy).
- A number indicating the sample, where samples are numbered sequentially, starting with 1, and contain no other intrinsic meaning.

Although primarily designed for large-scale biodiversity projects, agricultural species are also included (examples in Table 3). Adopting a widely used species identifier that is maintained by a dedicated entity should help guard against changes in species names.

**Table 3.** Formatting of identifiers using a sample of existing gene model identifiers to show improved consistency of names.

Original gene model ID	New assembly ID	New gene model ID
C01p010030.1_BnaDAR	ddBraNapu.DAR.1.0	ddBraNapu.DAR.1.1.01C.p010030
Glyma.01g000100.Wm82.a2.v1	drGlyMax.WM82.2.0	drGlyMax.WM82.2.1.01..g000100
Horvu_BARKE_1H01G000300.1	lpHorVulg.BARKE.1.0	lpHorVulg.BARKE.1.1.01..g000300
TraesCS3D02G273600	lpTriAest.CS.1.0	lpTriAest.CS.1.1.03D.g273600
Vitvi18g12230	drVitVini.PN40024.1.0	drVitVini.PN40024.1.1.18.g012230
Honeycrisp_HAP1_v1.0.031896	drMalDome.Honeycrisp.1.1.HAP1	drMalDome.Honeycrisp.1.1.3Hap1.g031896

Name format: <ToLID>.<variety>.<assembly-version>. <annotation-version>.<chr><opttional subgenome/haplotype>.<ENTITY>.<numeric ID>.

Stock/breed/subspecies/colony: The germplasm accession, variety, landrace, or breed identified in a human-readable form. The term should not contain spaces, and be as short as possible while remaining unambiguous within the species or clade.

Sequencing group: Although discussion participants did not give importance to indicating the assembly group or consortium, the authors have encountered different assemblies of the same cultivar produced by different groups, so we believe that this element should be included and will gain importance in the future.

Assembly version: A de novo reassembly of the same DNA, individual, or very closely related individual, or a significant improvement over an existing assembly is given a new version number. This could include an optional “v”, e.g. “v3”.

Optional: An optional term for needs that are not addressed above, for example, the haplotype for a phased assembly or secondary information for the Sample Short Name (e.g. location for an insect population). In a hypothetical example, a group named “TGMGP” sequences only the organelles of a peanut variety named ‘HighOil’. The assembly's name could be *drAraHypo.HighOil.TGMGP.1.0.organelles*. In another hypothetical example, for a haploid assembly of the apple variety ‘Gold Rush’ made by the same group, the primary haplotype could be named, *drMalDome.gr.TGMGP.1.Hap1* and the alternative haplotype *drMalDome.gr.TGMGP.1.Hap2*. While not recommended, a community may wish to append an identifier in an earlier nomenclature to ease the transition to a new naming scheme. For example, a subsequent assembly to *C_sonorensis_v2_redundans* could be named *idCulSono.KS.ABADRU.3.0.C_sonorensis_v3_redundans*. Care must be taken not to exceed the maximum allowed assembly names by INSDC databases.

### Gene model and transcript identifiers

**Fig. 2.**
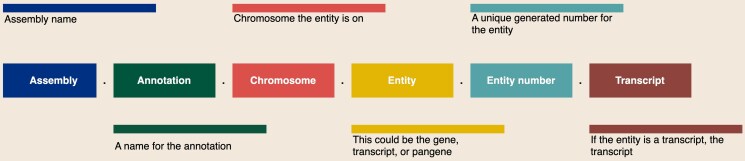
Gene model and transcript identifiers contain 6 fields, separated with periods: the assembly name, annotation name, chromosome (or scaffold), entity type, entity number, and if identifying a transcript, the transcript number.

The proposed format for gene model and transcript identifiers is shown in Fig. 2.

Assembly: Use the assembly name and version generated above as a prefix. This will unambiguously identify which version of an assembly the gene model comes from.

Annotation name: As multiple annotations may be calculated for the same assembly, an annotation designation is recommended.

Chromosome: Indicates chromosome with optionally-padded digits (e.g. “1” or “01”) followed by an optional subgenome and/or haplotype; for example, “01C” or “07hap1”.

Entity type: For example, *g* for gene, *p* for protein, *pan* for pan-gene, and *t* for transcript (see Table 2). This may be preceded by an optional character.

Entity number: A unique numeric identifier can be generated for each gene model within the genome. Six characters should be sufficient for numbering all gene models within an assembly. This number can be randomly generated or numbered sequentially. The latter is helpful for quickly identifying adjacent gene models.

Transcript: If the entity is a transcript, the transcript number. No assumption is made about which of multiple transcripts is designated as the canonical (i.e. representative) transcript for the gene mode.

The resultant genome nomenclature recommendation includes minimum information to create unambiguous yet human-readable identifiers for assemblies and gene models. We are aware that the resulting identifiers can be lengthy. However, identifiers of this length are within the accepted assembly name identifiers for the sequence archives (DDBJ, ENA, and SRA) and sequence processing/analysis software (e.g. BLAST and PathwayTools).

There are many advantages to submitting gene model annotations to an INSDC repository, which includes the assignment of unique identifiers that are guaranteed to be linked to metadata at the INSDC websites and in bulk download files. These identifiers can be used as primary identifiers in papers and record pages and downloads at community websites. Still, as they lack the human-readable characteristics of the proposed nomenclature, they may not be preferred by researchers. The 2 identifiers can co-exist and should be linked together, both at the INSDC repository and community databases, so that gene models can be found in either location with either identifier. When submitting to an INSDC repository, the identifiers recommended here could be indicated via the Dbxref parameter in the ninth (attributes) column in the submitted annotation's GFF file. However, this requires that the community database be recognized by the INSDC, which can be confirmed with this link: https://www.insdc.org/submitting-standards/dbxref-qualifier-vocabulary/.

To support the proposed nomenclature best practices, a companion command-line tool, the “AgBioData Assembly Name Generator”, has been developed. This Python-based tool, available at https://github.com/AgBioData/Genome-Assembly-and-Annotation-Nomenclature_WG, serves as a resource for the systematic creation of genome assembly and gene model identifiers. The tool adheres to the specifications outlined in this document and guides users through the process of generating file names tailored to the specific metadata attributes of reference genomes and is open source under the PDDL-1.0 license. The command-line tool provides a practical solution for users in genomics and related fields. The tool emphasizes simplicity and accessibility by minimizing dependencies. Additionally, for users who prefer containerization, the tool has been Dockerized, providing an alternative way to run it seamlessly in a controlled environment. Researchers are encouraged to explore this tool by cloning the Genome Assembly and Gene Model Identifier Tool GitHub repository (https://github.com/AgBioData/Genome-Assembly-and-Annotation-Nomenclature_WG), where thorough documentation and community support are provided. This tool and repository are designed to enhance collaborative efforts and promote effective integration into research workflows.

### Conclusion

Genome and gene nomenclature is often overlooked in genomic projects and data management plans, but intentionality in naming can prevent significant challenges later. A clear, standardized naming scheme benefits not only machine reliability and project organization but also facilitates long-term data reuse and interoperability. This paper highlights a set of generic rules that any project can follow, completely or in part, to simplify naming genome assemblies and provides ideas for how they might be adopted, along with examples of groups that have partially adopted the suggestions. The process of developing this standard involved: advocacy from a core group, engaging in conversation around needs in the broader community, carefully reviewing potential components, and facilitating adoption. Though achieving consensus is rarely straightforward, this process illustrates how intentionality and collaboration can lead to practical and widely accepted standards.

Data reuse is crucial in genomic research, as genome assembly and annotation data are often repurposed by other researchers. Consistent nomenclature simplifies the reuse of these datasets, enabling cross-species research, comparative analyses, and pan-genome studies that rely on multiple datasets. Inconsistent naming conventions hinder computational pipelines, while a unified approach not only aids in technical interoperability but ensures that gene model identifiers remain traceable to their respective assemblies. This, in turn, strengthens the integrity of downstream analyses and meta-analyses, enabling researchers to quickly assess the versions and assemblies discussed.

## Author notes

The U.S. Department of Agriculture is an equal opportunity lender, provider, and employer. Mention of trade names or commercial products in this report is solely to provide specific information and does not imply recommendation or endorsement by the U.S. Department of Agriculture.

## Supplementary Material

iyaf006_Supplementary_Data

## Data Availability

The AgBioData Assembly Name Generator is available at GitHub, https://github.com/AgBioData/Genome-Assembly-and-Annotation-Nomenclature_WG. Supplemental material available at GENETICS online.
