# Supplementary material for: Guidelines for gene and genome assembly nomenclature
Source: Genetics. 2025 Jan 15;229(3):iyaf006. doi: 10.1093/genetics/iyaf006 (PMC11912837; doi:10.1093/genetics/iyaf006)
Supplement: iyaf006_Supplementary_Data [file iyaf006_supplementary_data.zip › Supplemental_File_S1_GENETICS-2024-307538.pdf]

AgBioData Genome Assembly and Annotation Nomenclature Working Group survey

This survey is designed to
1) Gather feedback regarding genome assembly and gene model identifier naming preferences for AgBioData species
2) Explore metrics used for assessing genome assembly quality

Our goal is to understand community preferences regarding naming conventions and use of genome quality metrics, supporting the development of recommendations for our community.

A genome typically starts from a single individual of a given species, assembled from small sequencing reads. Subsequent increments that aim to improve genome quality and annotation (that is, where the genes are and what they encode) will incorporate more technologies and include more individuals, accessions/varieties/cultivars/breeds, as illustrated by this genome timeline.
How does one come up with a clear and unique identifier for each gene model from each of these genome assemblies?
Such is the goal of this survey

Your participation is voluntary and appreciated!

This survey should not take more than 15 min.

AgBioData

Toward enhanced genomics, genetics, and breeding research outcomes through standardization of practices and protocols across agricultural databases

Genome timeline

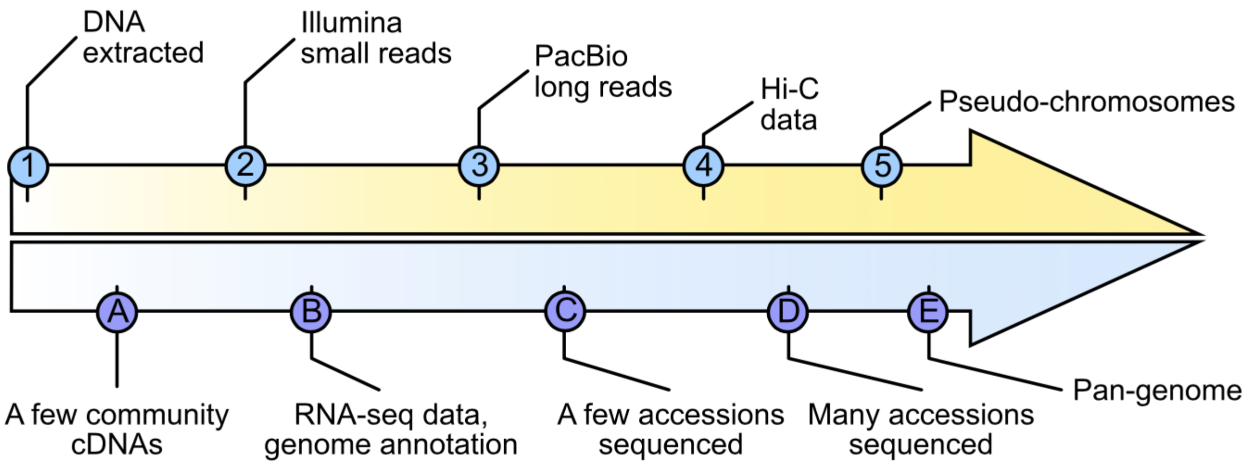

1. Please indicate using the numbers and letters on the genome timeline your current status of your sequencing project. E.g 2 & C

2. Email (optional):

Genome assembly nomenclature

This section focuses on identifiers for **genome assemblies** e.g.

- ARS-UCD1.2 (*Bos taurus*, cow)
- IRGSP-1.0 (*Oryza sativa*, rice)
- MorexV3_pseudomolecules_assembly (*Hordeum vulgare*, barley)
- Zm-B73-REFERENCE-NAM-5.0 (*Zea mays*, maize)

3. Which components should a genome assembly identifier include:

Check all that apply.

- [ ] Species name or identifier
- [ ] Variety / line / accession / individual identifier
- [ ] Genome assembly version
- [ ] Group providing the assembly
- [ ] Group providing the annotation (if different from above)
- [ ] Characters to space the components for readability e.g. dot, dash or underscore
- [ ] Other: ______________________

4. Based on your answers above, can you construct an example genome assembly identifier using all of those components in your preferred order?
e.g. ABCD_Brap_v1.01 (group, species, assembly version, annotation version)

____

Gene model identifiers

This section focuses on **identifiers for gene models** e.g.

- C01p010030.1_BnaDAR (*Brassica napus*, rapeseed)
- Glyma.01g000100.Wm82.a2.v1 (*Glycine max,* soybean)
- Vitvi18g12230 (*Vitis vinifera*, grape)
- Zm00001ea036589 (*Zea mays*, maize)

5. For your **main species of interest**, is there a gene model nomenclature system defined? Can you provide an example? e.g. Zm00001ea036589

____

____

____

____

____

6. If you have provided a gene model nomenclature system example, which features do you like / dislike?

____

____

____

____

____

7. If you were to annotate a genome for a species where other annotated genomes are already available, would you like to develop your own independent gene model identifiers or assign identifiers based on the existing annotations? e.g. gene000001 in assembly A and assembly B would be homologues

Mark only one oval.

- ◯ Unique to your assembly
- ◯ Homologues with related names

8. Should gene model identifiers be:

Mark only one oval.

- ◯ Human readable (i.e. confer information about the gene model)
- ◯ Machine readable (e.g. a numeric representation)
- ◯ Ideally both

9. Which components should a gene model identifier include:

Check all that apply.

- [ ] Species name or identifier
- [ ] Variety / line / accession / individual identifier
- [ ] Genome assembly version
- [ ] Genome annotation version
- [ ] Chromosome
- [ ] Sub-genome (e.g. for polyploids)
- [ ] Unique numeric identifier e.g. 00000123
- [ ] Entity e.g. 'g' for genes, 'p' for pangenes, 't' for transcript
- [ ] Gene annotation version (if different from genome annotation version)
- [ ] Group who provided the annotation
- [ ] Characters to space the components for readability e.g. dot, dash or underscore
- [ ] Other: ______________________________

10. Based on your answers above, can you construct an example gene model identifier using all of those components in your preferred order? e.g. Glyma.01g000100.Wm82.a2.v1 (species, chromosome, entity, numeric identifier, variety, assembly version, annotation version)

____

11. Do you have any other comments about genome assembly or gene model identifiers?

____

____

____

____

____

Genome quality metrics

This final section focuses on **metrics used to assess genome quality**

12. Do AgBioData databases provide adequate assembly and annotation quality metrics?

Mark only one oval.

- ⬭ Yes
- ⬭ No
- ⬭ Don't know

13. Which of the following metrics would you like to use to help you gauge genome quality?

Check all that apply.

- ☐ N50, L50 (length/contiguity statistics with respect to assembly size)
- ☐ NG50, LG50(length/contiguity statistics with respect to known/estimated genome size)
- ☐ BUSCO (assembly/annotation completeness for specified set of conserved genes)
- ☐ genome LTR Assembly Index (LAI)(assembly continuity using LTR retrotransposons)
- ☐ Other: ____________________

14. Do you have any other comments about genome metrics you would like ?

Thank you for your participation!

This content is neither created nor endorsed by Google.

Google Forms

AgBioData Genome Assembly and Annotation Nomenclature Working Group survey

16 responses

Please indicate using the numbers and letters on the genome timeline your current status of your sequencing project. E.g 2 & C

Copy

16 responses

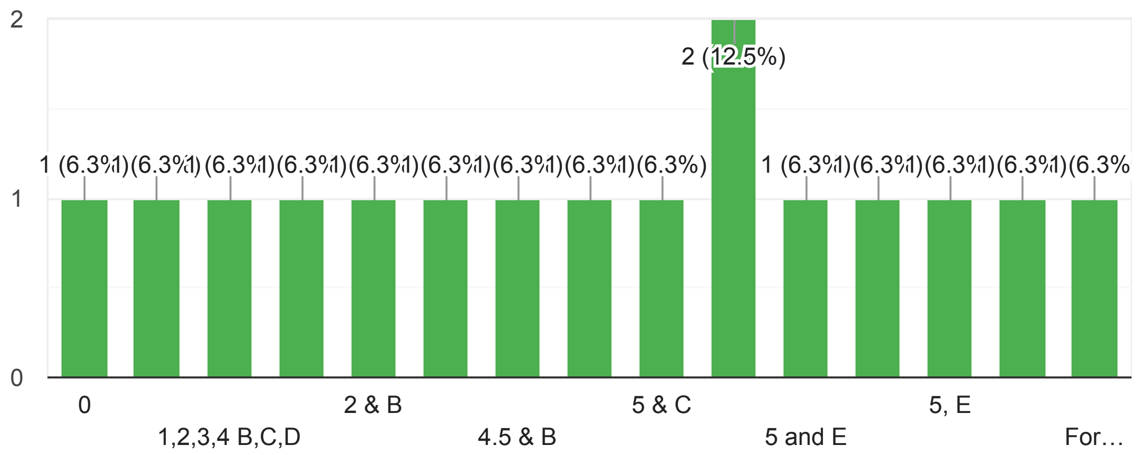

Which components should a genome assembly identifier include:

Copy

16 responses

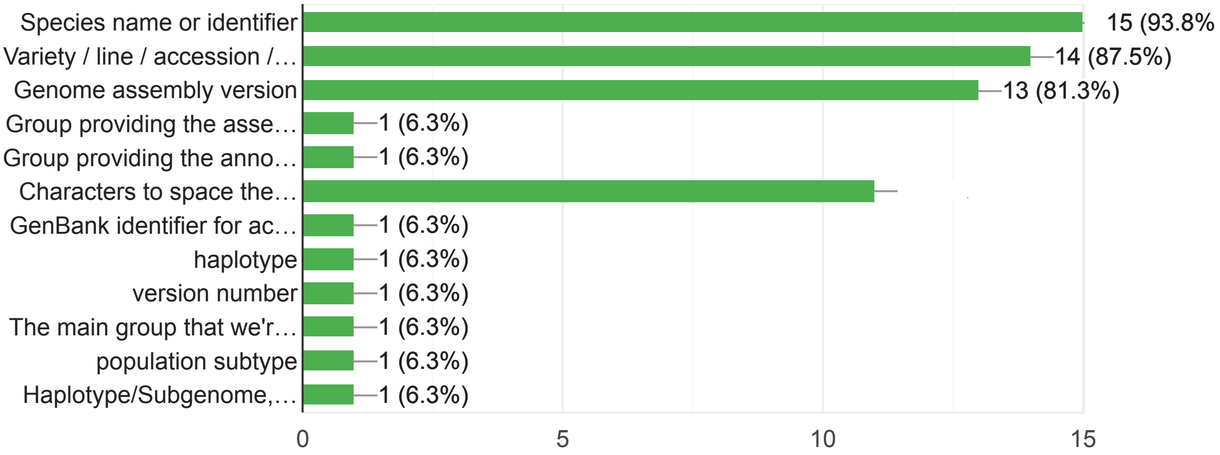

Based on your answers above, can you construct an example genome assembly identifier using all of those components in your preferred order? e.g. ABCD_Brap_v1.01 (group, species, assembly version, annotation version)

14 responses

Bdistachyon20180118 (common name), GCA_002892335.1(official name), see: https://www.ncbi.nlm.nih.gov/datasets/docs/v2/reference-docs/gca-and-gcf-explained/----all metadata about the assembly should be in the GCA file

Qalba_4.2.0.HapA_g6987 is an example of what we are currently using, its already too long and not enough information.

Species_GenomeAssemblyVersion_AnnotationVersion_ID

AraDur-K1000-2.0

iyBomHunt1.1 (see other examples here: https://www.ncbi.nlm.nih.gov/assembly?LinkName=bioproject_assembly_all&from_uid=555319)

Sh_Blk_v1.01 (species_variety_assembly version.annotation version)

Ppap_Isr_v1.01

Arath_v1.01

Group_PopulationType_GenusSpecies_Variety_V1.0

Arath.col0.v1

Maldo.hc.v1a1 (genus/species.variety.version/annotation)

Species-InfraName-Assembly_version

Brap_xxxxxx_V1.01

Ath_Col0_v1

Gene model identifiers

For your **main species of interest**, is there a gene model nomenclature system defined? Can you provide an example? e.g. Zm00001ea036589

16 responses

<1 letter genus><2 letter species><2 letter subspecies>version_chr_gxxxxx.1 (Macma4_01_g00030.1)

Yes. At1g01010

No.

No

Yes. See example above.

Our assemblies are annotated by NCBI's RefSeq database, and uses their accession numbers

For Brassicas, this is defined by the species coordination committee so standard across Brassica species

Sh1_v1.01.g00001 (for transcripts- Sh1_v1.01.g00001.t1, Sh1_v1.01.g00001.t2, ...)

PPAI000001

Phavu.10g0688767

Os01G0101700 or SAHI_SH5G0138257000

no

Maldo.hc.v1a1.ch10A.00001.t1

N/A

Hscxxxxx (Genus:"H", Species:"sc", scaffold/chromosome:the first"x", identifier:"xxxx")

MDXXGXXXXXXX

If you have provided a gene model nomenclature system example, which features do you like / dislike?

13 responses

difficult to deal with assembly for the same subspecies or species, not ideal for hybrids (and polyploids), ok for assembly version but not multiple annotations

like: simple

Arabidopsis and Phytozome do a good job of unique names and moving annotations over between versions. We are building pangenomes, not using other people's yet, so I think my opinion will change as we get deeper into that.

Identifiers are quite long.

like to have- Species, chromosome number, assembly and annotation version, gene id

I like the PP (denotes species)

Easy to understand

Prefixes suggesting the source, gene/transcript, chromosome#, potential order on the chromosome.

We do not have a example, but I'd like to include information that will be helpful in comparative genomic analysis.

I like this one because I made it

I don't care as long but it needs to be short, unique amongst all species, conistent in the assembly and backwards compatible between assemblies.

I would like to see the genes labelled in the numerical order by the position on chromosomes but want to keep it simple

I dislike that it is all one word, and that the other apple genome is MDOXXGXXXXXXX which looks almost the same except that the genomes do not correspond to each other

If you were to annotate a genome for a species where other annotated genomes are already available, would you like to develop your own independent gene model identifiers or assign identifiers based on the existing annotations? e.g. gene000001 in assembly A and assembly B would be homologues

Copy

15 responses

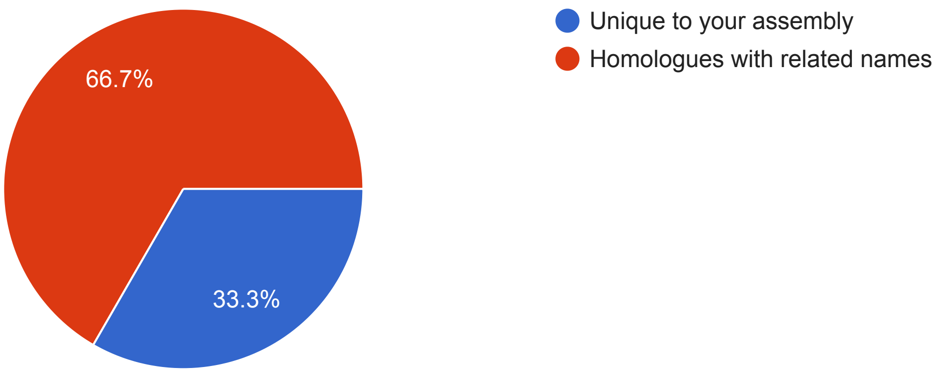

Should gene model identifiers be:

Copy

16 responses

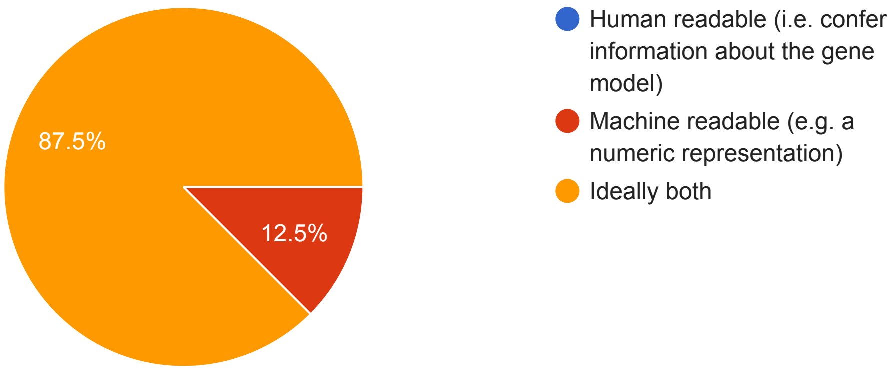

Which components should a gene model identifier include:

15 responses

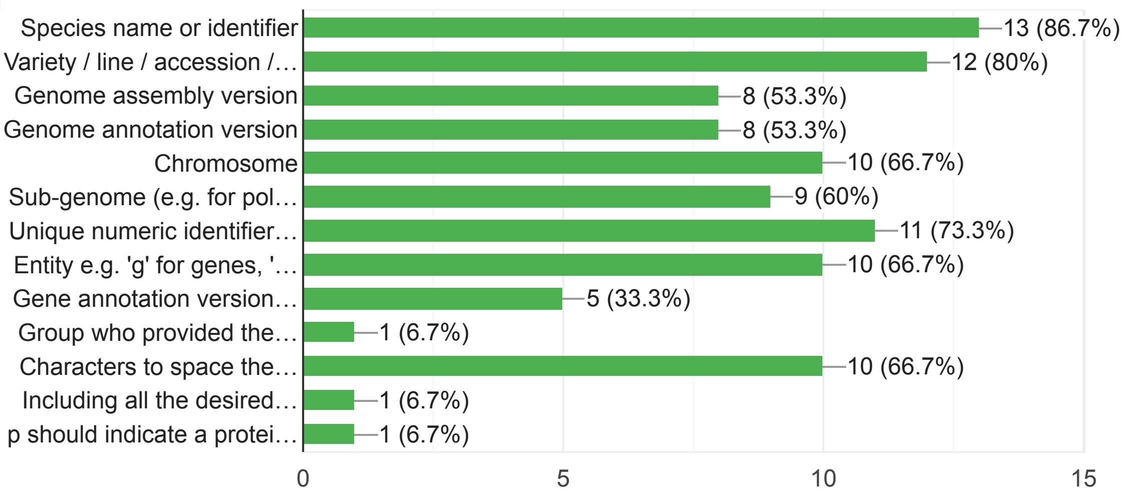

Based on your answers above, can you construct an example gene model identifier using all of those components in your preferred order? e.g. Glyma.01g000100.Wm82.a2.v1 (species, chromosome, entity, numeric identifier, variety, assembly version, annotation version)

11 responses

MacmaA4v1_cultivar_01_g00030.1

zero padded 9 digit id, 000000001

Not really, this is incredibly difficult.

AraDu2.0.00430, AraDu2.0.00430.t02, AraDu2.0.00430.p02

Sh1_Blk_v1.01_AB.g00001.01 (SpeciesChr#_variety_assembly version.annotation version_subgenome.gene identifier.gene annotation version)

ABCD_Brap_v1.01

SAHI_SH5G0138257000

Glyma.Wm82.hapA.01g123000

Maldo.hc.v1a1.ch10A.00001.t1 (genus/species.variety.version/annotation,chromosome/haplome.locus.entity)

Glyma01000100_Wm82.v1

MD_XX_XXXXXXX

Do you have any other comments about genome assembly or gene model identifiers?

10 responses

subgenomes can be useful for polyploids but information on ploidy too

Along with developing guidelines for naming genome assemblies/genes is newly sequenced genomes, guidelines on how to adapt/align existing nomenclature for already sequenced/studied species would be welcome.

Yes,this idea of "assign identifiers based on the existing annotations" is really great theoretically but nearly impossible in reality. we've been using GENESPACE to get robust orthologs between assemblies but even that leaves a lot of genes out due to structural variation, misassemblies, misannotations, etc. I think naming genes independently then grouping via a pangene is our best bet, as the pangenes will certainly change over time as the assemblies get better and more individuals are added. I'm also concerned that pangene/pangenome is such a loose concept. My original idea of this was working across individual genotypes within a species or across all interbreeding species (for example in a hybrid breeding program). IE within a population that can intercross. But now lots of people are publishing pangenomes at the genus or family level. Should those really be the same? I feel like we already have sufficient paralog/ortholog/homolog/orthogroup concepts for those broader studies and it would be helpful if the community could rally around a more stringent definition of a pangenome/pangene (but I realize this is unlikely!)

I think it's best to leave metadata out of accession numbers/identifiers - I know users like the metadata, but then they get attached to the identifier. NCBI RefSeq's accession system avoids this (but may not be as user friendly).

best to agree a standard among the community, some communities have this already

No

Create a Pan-gene catalog with designated IDs. Everyone used these to map their annotation and fill in the blanks by depositing the unique and isoforms variation back to atlas.

I would love to assign my gene identifiers based on the existing annotations, but without an existing high quality reference annotation (which is likely to be true in a lot of ag species), this seems infeasible.

no

Unfortunately, gene locus identifiers with all of that information become very long. Maybe genes need multiple names (aliases). One for the full name when comparing across species and a shorter version for within the genome. If gene naming is backwards compatible across versions then as long as the version is provided genes can change and referred to consistently across assemblies for the same genome.

Do AgBioData databases provide adequate assembly and annotation quality metrics?

16 responses

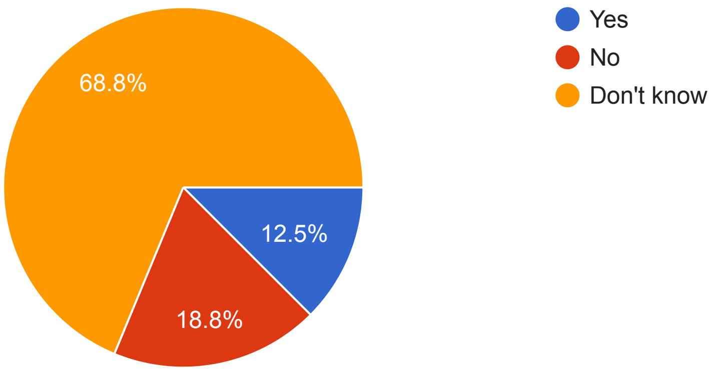

Which of the following metrics would you like to use to help you gauge genome quality?

16 responses

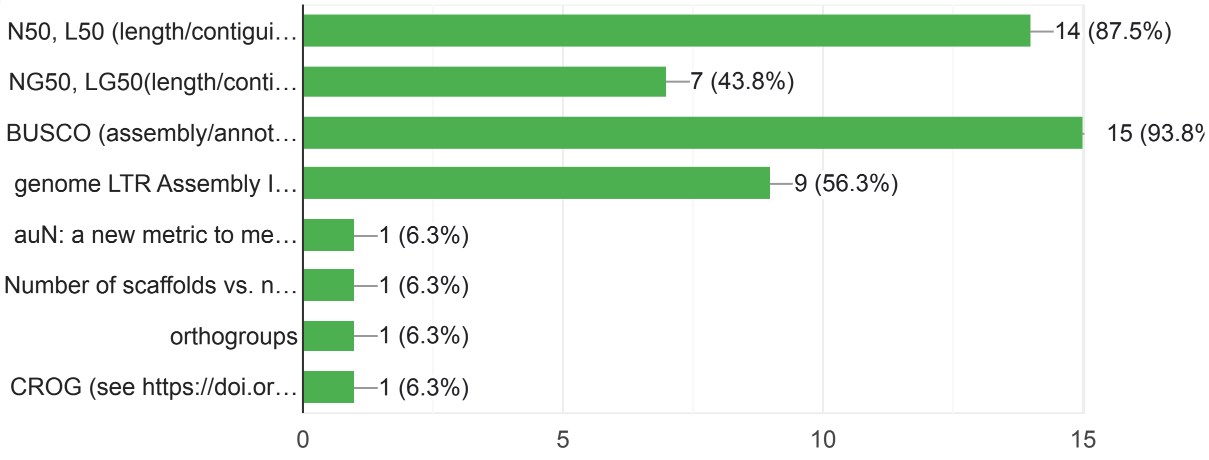

Do you have any other comments about genome metrics you would like ?

8 responses

Busco is nice but there are new approaches to check a wider number of gene families for completeness

Like to see stats for both contigs and scaffolds

I don't know whether all AgBioData databases provide adequate assembly and annotation quality metrics - I know that our database doesn't.

Most don't give a good representation of quality, data type and assembly method are good indicators, A PacBio HiFi assembly will be better than an Illumina assembly for example.

No

no

There needs to be some metric to assess quality of gene annotations. BUSCO can help identify coverage but some tools can predict 100K gene models and BUSCO doesn't catch that. It may give some folks a false sense of security to see a high BUSCO score but underneath the gene models are in highly repetitive regions or have weird splice-variant models (e.g. side-by-side) that make little sense.

I just want to comment that I am frustrated I can no longer access a ftp site on GDR. I know ftp is going out of fashion, but accessing it through clicking through 20 buttons is annoying when before I could just click on the directory I wanted.

Thank you for your participation!

This content is neither created nor endorsed by Google. Report Abuse - Terms of Service - Privacy Policy

Google Forms
