# Supplementary material for: Guidelines for gene and genome assembly nomenclature
Source: Genetics. 2025 Jan 15;229(3):iyaf006. doi: 10.1093/genetics/iyaf006 (PMC11912837; doi:10.1093/genetics/iyaf006)
Supplement: iyaf006_Supplementary_Data [file iyaf006_supplementary_data.zip › Supplemental_File_S2_GENETICS-2024-307538.pdf]

Supplemental File S2. Does the Proposed Naming Guideline Enable FAIR?

Findability Checklist

- Ensure genome assemblies are easy to find with clear and unambiguous naming.
  - Use of the Tree Of Life species id (ToLID) makes the primary information readily available, this is followed by further information to identify the individual that was sequenced.
- Include minimal metadata within names for easy filtering of data objects.
  - The naming scheme separates metadata fields so they can be identified
- Names should be easily distinguishable at a glance.
  - Species name, sequencing group, and the version are all readily available
- Maintain unique naming to avoid confusion.
  - While there exists a small possibility of two genome assemblies having the same name, it is unlikely due to the recommended metadata fields.

Accessibility Checklist

- Document the naming conventions and rules and make them easily accessible.
  - The github and this article document the convention.
- Consider implementing validation tools for the naming process.
  - A validation tool was written for the naming scheme as is available here: https://github.com/AgBioData/Genome-Assembly-and-Annotation-Nomenclature_WG
- Include provenance data within the name.
  - Several fields document provenance data in the proposed naming scheme
- Use intuitive and consistent naming rules for easier handling.

Interoperability Checklist

- Make names human and machine-readable.
  - Field separators and locations are formalized for machine-readability
- Formalize the naming scheme to ensure it is machine-readable and enables interoperability.
  - This document and the validation tool above formalize the naming scheme

Reuse Checklist

- Design names to be as informative as possible without being overly complex.
  - Effort was taken to minimize the number and length of fields in the proposed naming schemes.
- Incorporate references to relevant protocols within names.
  - The naming scheme utilizes ToLID and standard versioning practices from software engineering.
- Adhere to community practices.
  - The naming scheme has some flexibility to incorporate other community practices through the optional field, utilizes the ToLID naming convention, and tries to limit disruption to existing naming schemes.
